# Dietary Phospholipids Alleviate Diet-Induced Obesity in Mice: Which Fatty Acids and Which Polar Head

**DOI:** 10.3390/md21110555

**Published:** 2023-10-25

**Authors:** Lingyu Zhang, Jiaqin Mu, Jing Meng, Wenjin Su, Jian Li

**Affiliations:** 1 College of Ocean Food and Biological Engineering, Jimei University, Xiamen 361021, China; 18659577995@163.com (J.M.);; 2 College of Food Science and Engineering, Ocean University of China, Qingdao 266003, China; 3 National & Local Joint Engineering Research Center of Deep Processing Technology for Aquatic Products, Xiamen 361021, China; 4 Jining Institute for Food and Drug Control, Jining 272113, China

**Keywords:** phospholipids, obesity, fatty acid composition, headgroups, lipid metabolism

## Abstract

The weight loss effects of dietary phospholipids have been extensively studied. However, little attention has been paid to the influence of phospholipids (PLs) with different fatty acids and polar headgroups on the development of obesity. High-fat-diet-fed mice were administrated with different kinds of PLs (2%, *w*/*w*) with specific fatty acids and headgroups, including EPA-enriched phosphatidylcholine/phosphatidylethanolamine/phosphatidylserine (EPA-PC/PE/PS), DHA-PC/PE/PS, Egg-PC/PE/PS, and Soy-PC/PE/PS for eight weeks. Body weight, white adipose tissue weight, and the levels of serum lipid and inflammatory markers were measured. The expression of genes related to lipid metabolism in the liver were determined. The results showed that PLs decreased body weight, fat storage, and circulating lipid levels, and EPA-PLs had the best efficiency. Serum TNF-α, MCP-1 levels were significantly reduced via treatment with DHA-PLs and PS groups. Mechanistic investigation revealed that PLs, especially EPA-PLs and PSs, reduced fat accumulation through enhancing the expression of genes involved in fatty acid β-oxidation (*Cpt1a*, *Cpt2*, *Cd36*, and *Acaa1a*) and downregulating lipogenesis gene (*Srebp1c*, *Scd1*, *Fas*, and *Acc*) expression. These data suggest that EPA-PS exhibits the best effects among other PLs in terms of ameliorating obesity, which might be attributed to the fatty acid composition of phospholipids, as well as their headgroup.

## 1. Introduction

In recent decades, obesity has become a growing public health problem worldwide [1]. Importantly, there is a strong link between obesity and dyslipidemia, ectopic lipid accumulation, and chronic low-grade inflammation, which may lead to obesity-associated metabolic complications, such as type 2 diabetes (T2DM) and metabolic syndrome (MetS) [2]. Despite several efforts, the cause of obesity remains unclear [3,4], and the only effective treatment is gastric bypass surgery [5]. Therefore, finding new bioactive compounds to fight against obesity have received increasing interest [6,7].

A typical diet contains approximately 2–8 g of phospholipids (PLs), of which 10–40% (or 0.8 g) are egg-derived PLs [8]. Other food sources rich in PLs also include soy, dairy products, fish roe, and various seafoods [9]. Structurally, PLs are composed of a glycerol backbone esterified with two fatty acids, along with a phosphate that contains a polar headgroup. Based on the headgroup, dietary PLs can be categorized into different classes, primarily including phosphatidylcholine (PC), phosphatidylethanolamine (PE), and phosphatidylserine (PS). Moreover, the fatty acids (FAs) esterified to the sn-1 and sn-2 position of PL species differ significantly depending on the food sources. For example, egg PC consists primarily of palmitic acid (16:0) and oleic acid (18:1) at the sn-1 and sn-2 positions, respectively [8]. Soybean PLs are rich in n-6 polyunsaturated fatty acids (PUFAs) (mainly linoleic acid) [10]. Marine PLs are highly abundant in two n-3 PUFAs, docosahexaenoic acid (DHA) and eicosapentaenoic acid (EPA), which are preferentially bound at the sn-2 position of PLs [11]. Numerous lines of evidence support the association between the health efficacy of PLs and their structures, which include the different polar headgroups and fatty acid compositions [12,13,14].

Although PS is less abundant than PC is in dietary intake, there have been several studies regarding the neuroprotective effect of PS [15,16]. Recent findings suggest that dietary PS may have a similar effect on improving lipid metabolism to that of PC [17]. Moreover, growing interest has developed in marine-derived n-3 PUFAs due to their exceptional health benefits. Liu et al. found that EPA-PL was more effective than Soy-PL was in decreasing the lipid levels of liver and serum in high-fat-fed mice [18]. Another animal study demonstrated that EPA-PL had superior anti-obesity and lipid-lowering effects compared to DHA-PL [19]. Furthermore, a study comparing the effects of 12 different dietary phospholipids on the phospholipid profiles of organelles in the liver of NAFLD mice revealed that fatty acid composition of phospholipids may have a greater impact on the phospholipid composition of the organellar membrane than do the headgroups [13]. However, it is still unclear whether or not the fatty acid compositions of phospholipids have a stronger influence on lipid lowering compared to their headgroups. In this study, we aim to explore the influences of different kinds of PLs with specific fatty acids and headgroups (Soy-PC/PE/PS, Egg-PC/PE/PS, EPA-PC/PE/PS, and DHA-PC/PE/PS) on anti-obesity in high-fat-diet-induced obese mice. Moreover, we will investigate the possible underlying mechanisms by determining the expression of genes related to fatty acid metabolism and verifying the activating effects of different kinds of PLs on PPARs through a dual-luciferase report experiment.

## 2. Results

### 2.1. Effects of Different PLs on Growth Parameters in Mice

**Figure 1:**
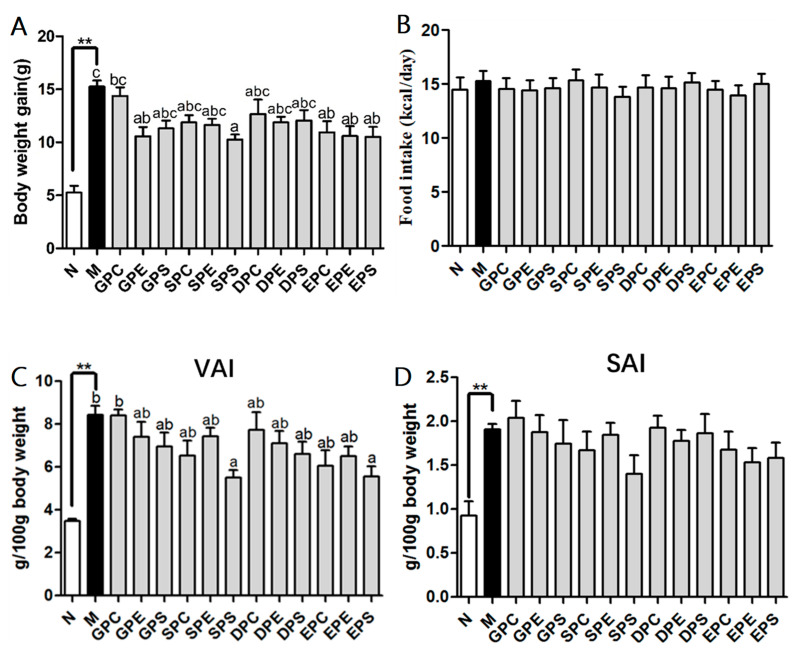
Effect of dietary phospholipids on body weight gain (**A**), energy intake (**B**), visceral adiposity index (VAI) (**C**), and subcutaneous adiposity index (SAI) (**D**) of mice. Data were given as mean ± SEM. The comparison of the control group (N) and the model group (M) was tested via Student’s *t*-test; ** *p* < 0.01. Letters indicate a significant difference at *p* < 0.05 among groups feeding a high fat diet (HFD), as determined via one-way ANOVA. Note: N (control), M (model), GPC (egg phosphatidylcholine), GPE (egg phosphatidylethnolamine), GPS (egg phosphatidylserine), SPC (soy phosphatidylcholine), SPE (soy phosphatidylethnolamine), SPS (soy phosphatidylserine), DPC (docosahexenoic acid-enriched phosphatidylcholine), DPE (docosahexenoic acid-enriched phosphatidylethnolamine), DPS (docosahexenoic acid-enriched phosphatidylserine), EPC (eicosapentaenoic acid-enriched phosphatidylcholine), EPE (eicosapentaenoic acid-enriched phosphatidylethnolamine), and EPS (eicosapentaenoic acid-enriched phosphatidylserine).

The body weight gain and food intake were measured to examine whether or not dietary phospholipids have different effects on the body weight of mice. After 8 weeks of feeding, the body weight of the model group was significantly higher than that of the control group, which suggested that the successful establishment of the obese mice model (Figure 1A). Different kinds of dietary phospholipids slowed down the increase in the body weight of high-fat-diet mice. Among these phospholipids (Soy-PC/PE/PS, Egg-PC/PE/PS, EPA-PC/PE/PS, and DHA-PC/PE/PS), phospholipids enriched in EPA were the most effective in decreasing body weight. The mice in the DHA phospholipid groups had a lower body weight than did the model group mice, although there was no statistically significant difference. Moreover, in the Soy-PS and Egg-PE groups, the body weight was significantly decreased compared to that in the model group, while there were no significant differences in body weight among the other phospholipid groups. As shown in Figure 1B, the food intake did not show significantly different among different groups. Table 1 showed that the model group exhibited a decrease in organ indexes compared to those of the control group, particularly in the liver and muscle indexes (*p* < 0.01). This decline may be attributed to the excessive body fat of the mice in the model group. After the administration of different phospholipids, there was an increasing trend in the muscle index of mice. Specifically, the muscle index significantly increased in the Soy-PS group and the EPA-PS group. Moreover, the high-fat diet increased lipid accumulation in white adipose tissues, including visceral white adipose tissue (VAT) and subcutaneous white adipose tissue (SAT), which further confirmed the success of the obesity model (Figure 1C,D). When mice were treated with phospholipids (PLs), varying degrees of reduction were observed in the weight of VAT and SAT. However, there was no significant difference in the weight of SAT among the different PL groups. It should be noted that EPA-PS and Soy-PS groups showed a superior reduction effect compared to that of other groups.

### 2.2. Effects of Different PLs on Lipid Profile in Serum of Mice

**Figure 2:**
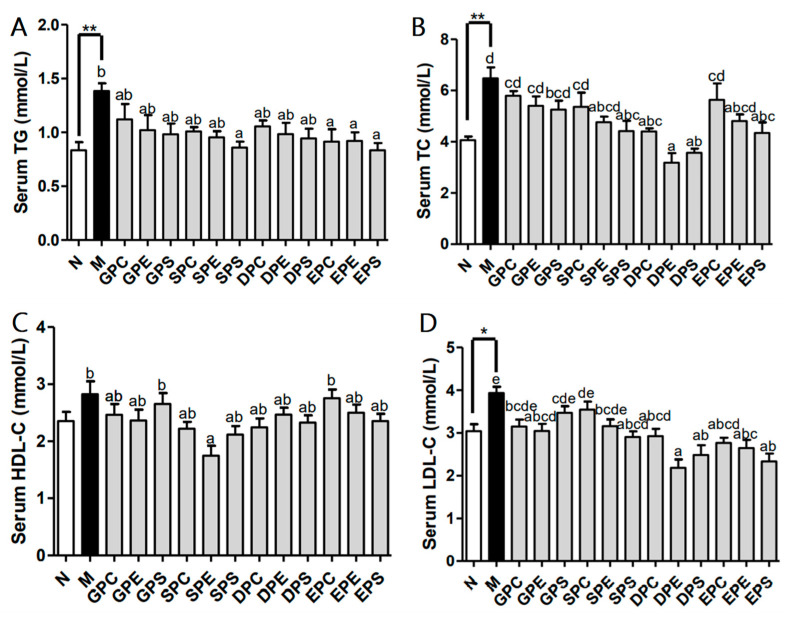
Effect of dieta0ry phospholipids on the serum lipid parameters of mice. The serum levels of triglycerides (TG), cholesterol (TC), high-density-lipoprotein cholesterol (HDL-C), and low-density-lipoprotein cholesterol (LDL-C) in mice were measured and are shown in (**A**), (**B**), (**C**), and (**D**), respectively. Data are given as mean ± SEM. The comparison of the control group (N) and the model group (M) was tested via Student’s *t*-test; ** *p* < 0.01. Letters indicate significant differences at *p* < 0.05 among groups feeding a high fat diet (HFD), as determined via one-way ANOVA.

Serum lipid levels can reflect the status of lipid metabolism in the body. In comparison to those of the mice in the control group, the levels of serum TG, TC, and LDL-C were significantly higher in the model group (*p* < 0.01) (Figure 2). There was a higher serum HDL-C level in the model group mice compared to that in the control group mice, although the difference was not statistically significant. Compared to the model group, dietary phospholipids led to a reduction in serum TG levels, with significant decreases observed in the Soy-PS group and EPA phospholipid groups. Treatment with phospholipids also resulted in a decrease in serum TC levels. Among the different phospholipid groups, the Soy-PS group, DHA phospholipid groups, and EPA-PS group dramatically reduced serum TC levels. However, egg phospholipid groups did not have a significant influence on serum TC levels. There were no significant differences in serum HDL-C levels among the different high-fat diet groups, except for the soy-PE treatment, which significantly decreased HDL-C levels in serum. Furthermore, compared to the model group, phospholipid treatment led to lower LDL-C levels. The Egg-PE group, Soy-PS group, and DHA and EPA phospholipid groups had significantly lower LDL-C levels than did the model group. The reduction in LDL-C levels in the EPA phospholipid groups may be related to the increase in HDL-C levels.

### 2.3. Effects of Different PLs on Inflammation Factors in Serum of Mice

**Figure 3:**
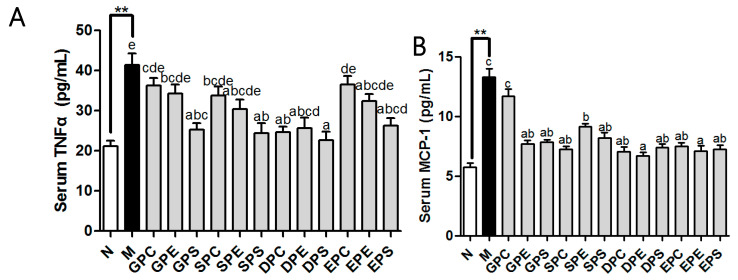
Effect of dietary phospholipids on the serum inflammation markers of mice. The serum levels of TNFα (**A**) and MCP-1 (**B**) were determined. Data were given as mean ± SEM. The comparison of the control group (N) and the model group (M) was conducted using Student’s *t*-test; ** *p* < 0.01. Letters indicate significant differences at *p* < 0.05 among groups fed a high fat diet (HFD) as determined via one-way ANOVA.

The inflammatory cytokines were measured in the serum. Figure 3 showed that the serum levels of TNF-α and MCP-1 were higher in the model group compared to those in the control group (*p* < 0.01). Dietary phospholipids reduced the serum level of TNF-α compared to that of the model group. Moreover, the mice in the Egg-PS group, Soy-PS group, DHA phospholipid group, and EPA-PS group had significantly lower levels of TNF-α than those in the model group. With the exception of Egg-PC, dietary phospholipids also significantly decreased the MCP-1 level compared to that in the model group.

### 2.4. Effects of Different PLs on mRNA and Protein Expression Associated with Lipid Metabolism in the Liver

**Figure 4:**
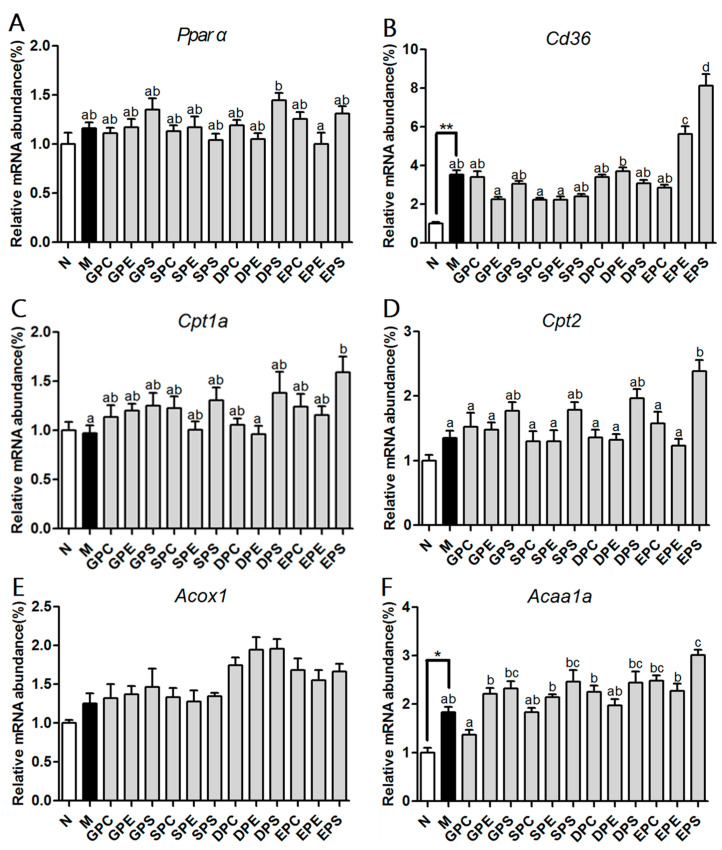
Effects of dietary phospholipids on hepatic fatty acid β-oxidation of mice. The mRNA expression of PPARα (**A**) and its target genes involved in fatty acid β-oxidation (**B**–**F**). Data were given as mean ± SEM. The comparison of the control group (N) and the model group (M) was conducted via Student’s t-test; * *p* < 0.05 and ** *p* < 0.01. Letters indicate significant differences at *p* < 0.05 among groups fed a high fat diet (HFD), as determined via one-way ANOVA.

**Figure 5:**
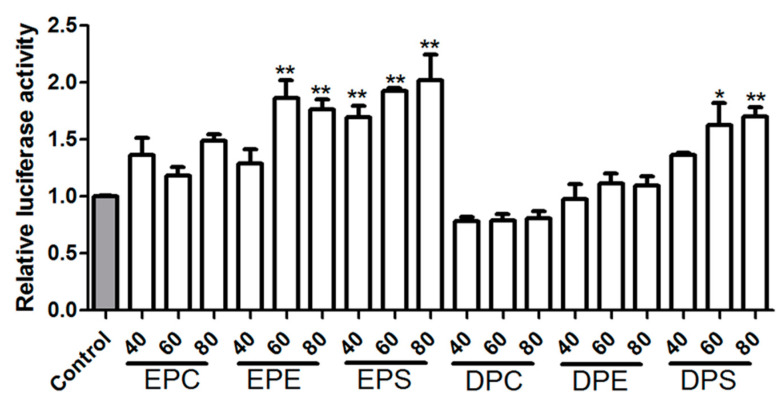
Transcriptional response of PPARs to EPA-PLs and DHA-PLs in HEK-293 cells. Cells were treated with 40, 80, and 100 mg/mL of EPA-PL for 24 h, and the control (gray bar) accepted no special treatment. Data were given as mean ± SEM. Significant differences between the treated and the control were tested via Student’s *t*-test; * *p* < 0.05 and ** *p* < 0.01.

**Figure 6:**
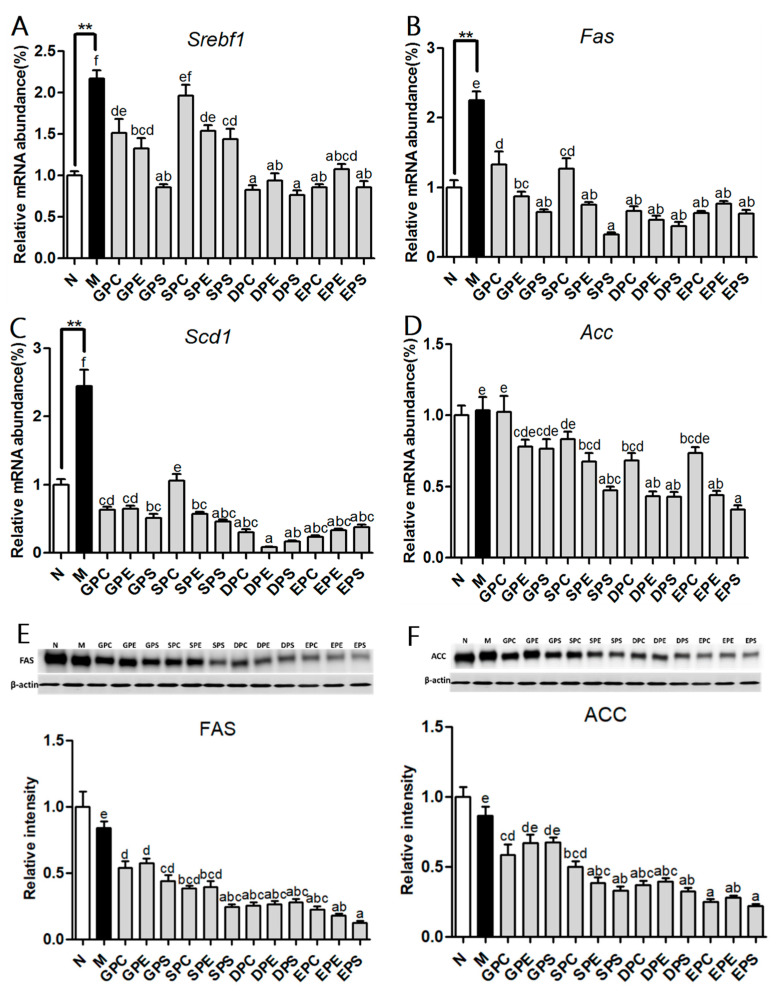
Effects of dietary phospholipids on hepatic fatty acid synthesis of mice. The mRNA expression of *Srebp1c* (**A**) and its target genes involved in lipogenesis (**A**–**D**). Western blot analysis of hepatic FAS, ACC, and β-actin protein (**E**,**F**). Data were given as mean ± SEM. The comparison of the control group (N) and the model group (M) was conducted via Student’s *t*-test; ** *p* < 0.01. Letters indicate significant differences at *p* < 0.05 among groups fed a high fat diet (HFD), as determined via one-way ANOVA.

The liver is a key player in lipid metabolism and other metabolic pathways [20]. Since the body weight and serum TG level were significantly suppressed by various phospholipids, the transcription levels of genes involved in lipid metabolism were determined (Figure 4). Peroxisome proliferation-activated receptor alpha (PPARα) is a crucial nuclear transcription factor that targets genes related to hepatic fatty acid β-oxidation [21]. The results showed that there were no significant differenc es in the mRNA expression levels of *Pparα* and *Acox1* among the phospholipid groups and the model group. However, compared to the model group, EPA-PE and EPA-PS significantly upregulated the expression of *Cd36* (Figure 4B). Additionally, the mRNA expression levels of *Cpt1a* and *Cpt2* were increased in all phospholipid groups, with a significant upregulation of *Cpt1a* in the EPA-PS group and a significant increase in *Cpt2* expression in all PS groups (Figure 4C,D). Compared to the model group, dietary phospholipids increased the expression levels of *Acaa1a* mRNA, while a significant increase was observed only in the EPA-PS group (Figure 4F). Furthermore, a dual-luciferase reporter gene assay was performed to investigate the activation of PPAR via the use of different concentrations of phospholipid-enriched EPA and DHA. As shown in Figure 5, EPA-PE, EPA-PS, and DHA-PS activated PPAR in a dose-dependent manner. Specifically, EPA-PS was able to activate PPAR at the lowest concentration of 40 μg/mL. These findings are consistent with that of the upregulation of PPARα target genes observed in mice of the EPA-PS group. Lipid homeostasis is regulated via the synthesis and catabolism of lipids. The expression of genes related to lipid synthesis were further measured (Figure 6A–D). Compared to the control group, a high-fat diet significantly upregulated the levels of Srebf1 and its target genes, *Fas* and *Scd1*, in the liver of the model group mice. Compared to the model group, the mRNA expressions of *Srebf1*, *Fas*, *Scd1*, and *Acc* were all decreased to varying degrees in the mice of the phospholipid groups. Protein levels of FAS and ACC were analyzed via Western blot analysis (Figure 6E,F), which showed consistent changes with the mRNA expression levels of the studied genes.

### 2.5. Interaction Effects between Fatty Acids and Polar Head Groups

The observations are summarized in Table 2, which was used to evaluate the key components in PLs that contribute to their anti-obesity properties. The results revealed that the fatty acid composition of PLs modified 14 indices, while polar headgroups of PLs influenced 15 indices. This suggested that the lipid-lowering effects of PLs are dependent on both their polar headgroups and fatty acid composition. Furthermore, significant interactions were observed between fatty acids and polar heads for five indices, including serum MCP-1 levels and the mRNA expression of lipolysis and lipogenesis genes (*Cd36*, *Acaa1a*, *Srebf1*, and *Scd1*).

## 3. Discussion

In the current study, we compared the anti-obesity effects of 12 kinds of phospholipids with varying fatty acids and polar headgroups and linked the underlying mechanisms to chronic inflammation and lipid metabolism. Among all phospholipids, EPA-enriched phospholipids and PS were the most effective in reversing HFD-induced obesity and hyperlipidemia in mice. These improvements were accompanied by an increase in fatty acid β-oxidation and a decrease in lipid synthesis. Moreover, DHA-enriched phospholipid groups, along with PS groups, exhibited the strongest anti-inflammatory effects compared to other phospholipid groups.

The present study revealed varying degrees of weight loss in mice across different phospholipid groups, indicating variations in the anti-obesity effects of these phospholipids. The health effects of the phospholipids are highly correlated with their fatty acid compositions. Shirouchi et al. compared the effects of egg-PC and phosphatidylcholine enriched with n-3 PUFAs (n3-PC) using OLETF rats [22]. The findings revealed that only n3-PC could reduce serum lipid levels, which were consistent with the results of our study, suggesting that EPA-enriched phospholipids exhibited superior effects on improving obesity compared to Egg-PLs. Moreover, the beneficial effects of n-3 fatty acids or other kinds of fatty acids were attributed in part to their storage form. Buang et al. performed a comparative study of PC and TG with similar fatty acid compositions, which demonstrated that only PC significantly reduced serum lipid levels in whey acid-induced SD rats [23]. Imaizumi et al. also found that soy-PE was more effective than soy-PC in reducing serum lipids [24]. This difference in efficacy might be due to the presence of the ethanolamine group in PE, which can alter the phospholipid composition of the lipoprotein surface membrane and consequently impact hepatic lipid metabolism. In the present study, consistent with the findings of previous studies, the mice of PE groups had lower blood lipid (TC; LDL-C) levels than those in PC groups.

Obesity is characterized by chronic low-grade inflammation, and adipose tissue is known to release many inflammatory factors [25]. Obese individuals most frequently display abnormal serum levels of inflammatory cytokines, such as TNF-α and MCP-1 [26]. A previous study has demonstrated a negative correlation between dietary choline intake and serum inflammatory factor levels [27]. The choline headgroup of PC is one of the possible reasons why egg-PC can decrease the levels of the inflammatory factors. The in vitro experiments showed that two types of PE molecules significantly inhibited platelet aggregation induced via PAF, an inflammatory mediator involved in chronic inflammation [28]. Phosphatidylserine (PS) is required for healthy nerve cell membranes and myelin, and it plays an important role in improving cell metabolism [15]. As a result, extensive research has been conducted to investigate the effects of PS on brain function and specific neurotransmitters in experimental animals [14,16]. Additionally, previous studies have demonstrated that soy-PS could reduce body weight, decrease adipocyte volume, and improve symptoms related to metabolic syndrome in obese rats [29]. Our results further support these results, as we found that PS was superior to PC in reducing serum lipid levels, and exhibited better effects in decreasing bodyweight and inflammation compared to both PC and PE.

The liver plays a key role in lipid metabolism. When energy intake increases, there is a disruption in hepatic lipid metabolism, resulting in the inhibition of lipoprotein secretion and abnormal lipid accumulation in the liver [20]. Our previous study confirmed that the fatty acid composition of dietary PLs had a greater impact on reducing hepatic lipid accumulation than did the headgroups in NAFLD mice [13]. Specifically, PLs containing EPA or DHA demonstrated better efficiency than did the PLs from soy or egg. Lipid metabolism involves several pathways, including fatty acid uptake, synthesis, and oxidation. Non-esterified fatty acids (NEFA) can enter hepatocytes through fatty acid transport proteins such as CD36 [20]. In the liver, these NEFAs then transfer into the mitochondria for β-oxidation or traffic into the nucleus to bind to the transcription factor and regulate gene expression. PPARα is a transcription factor that plays a major role in lipid metabolism by regulating the expression of numerous target genes, such as *Acox1* and *Cpt1* [21]. Moreover, PPARα is predominantly expressed in liver and brown adipose tissue, and other tissues with an active energy metabolism [21]. Different types of fatty acids are the most abundant ligands for PPARα. Our present data showed that EPA-enriched phospholipids can significantly increase the expression of PPARα target genes (*Cpt1a*, *Cpt2*, and *Acaa1a*) compared to other types of phospholipids. Previous studies have also found that EPA is a stronger activator of PPARα compared to alpha-linolenic acid (C18:3) [30]. The strongest activation of PPARα is observed when feeding fish oil. Additionally, although fatty acids with 22 carbon atoms have a weak ability to activate PPARα, they can be metabolized into 20-carbon-atom fatty acids in cells, which can then activate PPARα [31].

Several studies have investigated the activation of PPARα by specific phospholipid species, including phosphatidylcholines PC (16:0/18:1) or PC (18:0/18:1) [32]. Although PC (16:0/18:1) is one of the most abundant phospholipid species in egg-PC [8], a study by Cohn et al. found that feeding with egg-PC for 3 weeks did not upregulate the levels of PPARα target genes in the liver [9]. Egg-PC might decrease circulating lipid levels by reducing the intestinal absorption of lipids, rather than regulating PPARα [8]. In addition, our results reveal that PS can significantly increase β-oxidation capacity compared to PC and PE through activating PPARα, suggesting that certain specific PS species may be more potent PPAR agonist, leading to enhanced β-oxidation.

The nuclear transcription factor *Srebp1c*, known for its role in regulating fatty acid synthesis, is a target of PUFAs control in the liver [33]. Numerous studies have reported the inhibition effect of PUFAs on the expression of *Srebp1c* and its downstream genes involved in fatty acid synthesis [34,35]. Our results demonstrated that EPA/DHA-PLs significantly downregulated the expression of lipogenic target genes compared to soy-PLs and egg-PLs, which is consistent with previous findings [18]. The influence of PUFAs on the regulation of fatty acid synthesis involves both transcriptional and post-transcriptional regulation mechanisms. Studies have shown that PUFAs can suppress the expression of *Fas*, *Acc*, and other lipogenic target genes through the post-transcriptional regulation of SREBP-1 [33,36]. Additionally, the polar headgroups of phospholipids also play a significantly role in the expression of genes related to fatty acid synthesis. Compared to PC and PE, dietary PS significantly inhibits the expression of fatty acid synthesis genes (*Fas*, *Scd1*, and *Acc*), suggesting that PS has a larger effect on inhibiting the expression of genes involved in lipogenesis.

## 4. Materials and Methods

### 4.1. Preparation of Phospholipids

EPA- and DHA-PC were obtained from sea cucumber (*Cucumaria frondosa*) and squid (*Illex argentinus*) roe, respectively, and the preparation procedures were performed as described in the literature [19]. Soy-PC with a purity of 95% was purchased from Avanti Polar Lipids, while egg-PC with a purity of 90% was purchased from Beijing lecithinchina Co., Ltd.(Beijing, China). In accordance with Hosokawa et al. [37], PEs and PSs were produced from PCs through PLD-mediated transphosphatidylation. The purity of various phospholipids was determined to be above 90% using thin-layer chromatography (TLC) plates and the molybdenum blue colorimetric methods.

### 4.2. Animals and Diets

All aspects of the animal care and experimental protocols were approved by the Ethical Committee of the College of Food Science and Engineering of the Ocean University of China. Six-week-old male C57BL/6J mice were purchased from Vital River Laboratory Animal Technology Co. (Beijing, China). After an acclimatization period of one-week, mice were randomly divided into 14 groups (*n* = 6 per group). Experimental groupings were as follows: the control group was fed a standard diet, the model group was fed a high-fat diet (HFD), and the PL groups were fed a HFD plus 2% of the corresponding PL. The formulations of the experimental feeds are shown in Table S1. The fatty acid compositions of the experimental diets used in the study were determined using the method outlined by Lou et al. [38]. Mice had free access to food and water, and were weighed every other day. The mice were fed in single cages and their feed residues were weighted daily. After 8 weeks, all mice were subjected to fasting for 12 h and then sacrificed. Blood samples were collected and serum was obtained via centrifugation at 1000× *g* for 15 min at 4 °C. Livers, adipose tissues, muscle, and other tissues were harvested from these mice, and stored at −80 °C for further analysis.

### 4.3. Analysis of Serum Parameters

Serum total cholesterol (TC), triglyceride (TG), LDL-cholesterol (LDL-C), and HDL-cholesterol (HDL-C) levels were determined using commercial kits (BioSino Biotechnology and Science Inc., Beijing, China). Serum concentrations of tumor necrosis factor-α (TNF-α) and monocyte chemoattractant protein 1 (MCP-1) were measured using commercial ELISA kits (Thermo Fisher Scientific, Waltham, MA, USA).

### 4.4. RNA Extraction and Quantitative Real Time PCR

Total hepatic RNA was extracted using Trizol Reagent (Invitrogen, Carlsbad, CA, USA) following the supplier’s instructions. RNA was reversed to cDNA using a random primer (TOYOBO, Osaka, Japan). The target genes were amplified using SYBR Green I Master Mix (Roche, Mannheim, Germany) in an iCycler iQ5 system (Bio-Rad Laboratories Inc., Hercules, CA, USA) with specific primers. The thermal conditions were as follows: 1 cycle of 95 °C for 10 min, 45 cycles of 95 °C for 15 s, 55–60 °C for 20 s, and 72 °C for 30 s. The primer list is given in Table S3. Relative gene expression was normalized to β-actin, and analyzed using the relative standard curve method.

### 4.5. Western Blot Analysis

Hepatic samples were lysed in RIPA lysis buffer (BiYunTian, Shanghai, China) following the manufacturers’ instructions. The protein concentrations were determined using a BCA Protein Assay kit (Beyotime Biotechnology, Beijing, China). Proteins were separated on 8% acryl amide gels and transferred to a PVDF membrane (Millipore, Billerica, MA, USA). Membranes were blocked for 2 h at RT in a 5% nonfat milk solution in Tris-buffered saline containing 0.5% Tween 20, and incubated with antibodies for 2 h. HRP detection was carried out using ECL plus reagent (Engreen, Beijing, China) in accordance with the manufacturer’s instructions. Western blotting was used to determine the expression of FAS (#3180S, Cell Signaling Technology (Beverly, MA, USA)) and ACC (#3676S, Cell Signaling Technology) in the liver. Protein loading was evaluated using β-actin antibody (sc-47778, Santa Cruz (Santa Clara, CA, USA)). The protein bands were quantified via band intensity and band area.

### 4.6. Luciferase Reporter Assay

The pGMPPAR-Lu reporter plasmid was obtained from Genomeditech Co., Ltd. (Shanghai, China). Human embryonic kidney (HEK) 293 cells were seeded on a 24-well plate and transfected with 450 ng of pGMPPAR-Lu and 50 ng of pRL-TK per well. After 24 h of transfection, the cells were treated with EPA-PC/PE/PS and DHA-PC/PE/PS (40, 60, or 80 µg/mL) for 24 h. Following cell lysis, luciferase activities were analyzed using Dual-Luciferase Reporter Assay System (Promega, Madison, WI, USA) in accordance with the manufacturer’s recommended procedure.

### 4.7. Statistics

Data were presented as the mean ± SEM. A two-tailed t-test was used to compare two groups, and one-way ANOVA with a post hoc Tukey test was used to compare multiple groups (SPSS version 19.0). A statistically significant result was defined as a *p*-value less than 0.05. Graphs were generated using the Prism 5 software (Graph-Pad Software, San Diego, CA, USA).

## 5. Conclusions

In conclusion, the present study demonstrates that dietary PLs were capable of ameliorating weight loss and fat accumulation in HFD-induced mice. Among the phospholipids with different fatty acid compositions, EPA-PLs showed more pronounced benefits in terms of weight loss. Furthermore, the anti-obesity and lipid-lowering effects of PS were superior to those of PC and PE. Mechanistic investigation revealed that EPA-PS reduced fat storage by activating PPARα and regulating genes related to lipid metabolism. The novelty in this work is that we investigated the anti-obesity effects of multiple phospholipids that vary in acyl-chain and polar headgroup composition in one study. Compared to soy phospholipids and egg phospholipids, marine phospholipids have a more unique fatty acid composition. These phospholipids, which contain specific fatty acids, such as EPA and DHA, could have a more significant impact on human health. Moreover, phospholipids products such as soy lecithin are a mixture of various phospholipids, and the production of specific phospholipid species (e.g., PS) deserves further attention.

## Figures and Tables

**Table 1:** Effects of different kinds of phospholipids on growth parameters in mice.

g/100 g Body Weight	N	M	GPC	GPE	GPS	SPC	SPE
liver	3.80 ± 0.08	3.02 ± 0.08 ^ab^ **	2.69 ± 0.14 ^a^	3.02 ± 0.14 ^ab^	3.15 ± 0.06 ^ab^	3.06 ± 0.12 ^ab^	3.01 ± 0.07 ^ab^
kidney	1.24 ± 0.03	1.10 ± 0.02	1.12 ± 0.04	1.14 ± 0.06	1.14 ± 0.04	1.17 ± 0.03	1.12 ± 0.04
heart	0.59 ± 0.03	0.50 ± 0.02	0.49 ± 0.03	0.48 ± 0.02	0.47 ± 0.02	0.50 ± 0.04	0.47 ± 0.03
lung	0.60 ± 0.01	0.47 ± 0.03	0.49 ± 0.06	0.44 ± 0.02	0.51 ± 0.03	0.50 ± 0.03	0.49 ± 0.03
spleen	0.22 ± 0.00	0.22 ± 0.02	0.23 ± 0.02	0.22 ± 0.02	0.22 ± 0.01	0.23 ± 0.02	0.22 ± 0.01
thymus	0.18 ± 0.01	0.16 ± 0.01	0.16 ± 0.01	0.17 ± 0.00	0.17 ± 0.01	0.16 ± 0.01	0.16 ± 0.01
muscle	1.20 ± 0.01	0.96 ± 0.03 ^a^ **	1.00 ± 0.01 ^ab^	1.01 ± 0.04 ^abc^	1.06 ± 0.05 ^abc^	1.04 ± 0.03 ^abc^	1.00 ± 0.03 ^ab^
g/100 g body weight	SPS	DPC	DPE	DPS	EPC	EPE	EPS
liver	3.39 ± 0.14 ^bc^	3.10 ± 0.20 ^ab^	2.90 ± 0.07 ^ab^	3.16 ± 0.38 ^ab^	3.12 ± 0.12 ^ab^	3.35 ± 0.12 ^bc^	3.15 ± 0.15 ^ab^
kidney	1.21 ± 0.04	1.10 ± 0.07	1.12 ± 0.06	1.16 ± 0.15	1.15 ± 0.03	1.11 ± 0.01	1.24 ± 0.04
heart	0.58 ± 0.04	0.45 ± 0.04	0.48 ± 0.01	0.47 ± 0.06	0.51 ± 0.04	0.50 ± 0.03	0.55 ± 0.03
lung	0.56 ± 0.02	0.47 ± 0.03	0.45 ± 0.02	0.49 ± 0.06	0.48 ± 0.04	0.52 ± 0.02	0.53 ± 0.03
spleen	0.22 ± 0.02	0.27 ± 0.06	0.23 ± 0.02	0.23 ± 0.03	0.25 ± 0.02	0.23 ± 0.02	0.28 ± 0.02
thymus	0.19 ± 0.01	0.19 ± 0.01	0.17 ± 0.01	0.17 ± 0.03	0.17 ± 0.01	0.15 ± 0.01	0.17 ± 0.01
muscle	1.14 ± 0.03 ^bc^	0.99 ± 0.03 ^ab^	1.03 ± 0.06 ^abc^	1.05 ± 0.05 ^abc^	1.04 ± 0.03 ^abc^	1.09 ± 0.02 ^abc^	1.11 ± 0.02 ^bc^

Note: ** *p* < 0.01; significant difference compared to the N group determined via Student’s *t* test. Different letters indicate significant differences at *p* < 0.05 among high fat diet groups as determined via one-way ANOVA (Tukey’s test).

**Table 2:** The effect of fatty acids and polar head groups on obesity.

*p*-Value	FAs	Polar Head Groups	FAs × Polar Head Groups
Growth parameters			
Body weight gain	NS	0.04	NS
vWAT	0.01	0.025	NS
Serum lipid profile			
TG	NS	NS	NS
TC	<0.001	0.002	NS
HDL-C	0.002	NS	NS
LDL-C	<0.001	0.002	NS
Serum inflammation markers			
TNF-α	<0.001	<0.001	NS
MCP-1	<0.001	0.014	<0.001
Gene related to lipid oxidation			
*Cd36*	<0.001	<0.001	<0.001
*Cpt1a*	NS	0.006	NS
*Cpt2*	NS	<0.001	NS
*Acaa1a*	<0.001	<0.001	0.013
Gene related to lipid synthesis			
*Srebf1*	<0.001	<0.001	0.007
*Fas*	<0.001	<0.001	NS
*Scd1*	<0.001	<0.001	<0.001
*Acc*	<0.001	<0.001	NS
Protein related to lipid synthesis			
FAS	<0.001	0.001	NS
ACC	<0.001	NS	NS

Note: NS, not significant. Data were analyzed via two-way ANOVA followed by Tukey’s test.

## Data Availability

Data are contained within the article or Supplementary Materials.

## Supplementary Materials

The following supporting information can be downloaded at https://www.mdpi.com/article/10.3390/md21110555/s1. Table S1: Composition of the experimental diet; Table S2: Fatty acid composition of experimental diets; Table S3: Primer Information.
