# Supplementary material for: Dietary Phospholipids Alleviate Diet-Induced Obesity in Mice: Which Fatty Acids and Which Polar Head
Source: Mar Drugs. 2023 Oct 25;21(11):555. doi: 10.3390/md21110555 (PMC10672366; doi:10.3390/md21110555)
Supplement: Supplementary file 1 [file marinedrugs-21-00555-s001.zip › marinedrugs-2656154-supplementary.pdf]

Table S1. Composition of the experimental diet

Ingredients (g kg^{-1})	Normal	Model	Phospholipids
Corn Starch	650	200	200
Casein	200	200	200
Fructose	--	250	250
Powdered Cellulose	50	50	50
Mineral mix	35	35	35
Vitamin mix	10	10	10
DL-methionine	3	3	3
Choline bitartrate	2	2	2
Corn oil	50	50	46
lard	--	200	184
Phospholipid	--	--	20

Note: --, none added.

Table S2. Fatty acid composition of experimental diets

	Normal	Model	GPC	GPE	GPS	SPC	SPE	SPS	DPC	DPE	DPS	EPC	EPE	EPS
C14:0	1.27	2.07	1.29	1.29	1.73	1.43	1.27	1.51	1.81	1.93	1.89	1.71	1.77	1.67
C16:0	20.52	23.03	27.42	26.81	25.92	19.26	19.72	19.13	24.59	25.48	25.22	19.95	20.87	20.42
C16:1	--	1.87	1.23	1.26	1.33	0.71	0.63	1.38	1.46	1.40	1.10	1.69	1.24	1.60
C18:0	1.22	14.01	14.51	14.16	14.05	9.17	8.90	8.83	11.37	10.79	12.17	11.65	12.31	12.53
C18:1	30.18	36.14	31.41	31.88	32.94	23.99	23.52	23.51	29.62	28.38	28.83	32.53	31.92	32.77
C18:2	45.53	21.40	19.28	19.92	19.33	42.06	42.48	42.33	18.88	18.47	18.03	23.17	22.46	22.02
C18:3	1.28	0.80	0.53	0.60	0.66	3.38	3.48	3.31	0.63	0.61	0.60	0.77	0.68	0.72
C20:1	--	0.69	0.36	0.37	0.53	--	--	--	2.11	2.47	2.29	1.03	0.98	0.95
AA	--	--	3.06	2.94	2.79	--	--	--	--	--	--	--	--	--
EPA	--	--	--	--	--	--	--	--	2.06	2.74	2.56	7.20	7.32	7.12
DHA	--	--	0.91	0.77	0.73	--	--	--	7.47	7.73	7.31	0.31	0.44	0.19
SFA	23.01	39.10	43.22	42.26	41.69	29.86	29.89	29.47	37.77	38.20	39.28	33.31	34.96	34.63
MUFA	30.18	38.69	33.00	33.51	34.80	24.70	24.15	24.89	33.19	32.25	32.22	35.25	34.15	35.32
PUFA	46.81	22.20	23.78	24.23	23.51	45.44	45.96	45.64	29.04	29.55	28.50	31.44	30.89	30.05

Note: --, not detected.

Table S3. Primers Information

Target genes	Fw Primer Sequence (5′-3′)	Rv Primer Sequence (5′-3′)
Pparα	GTACGGCAATGGCTTTATCA	CAATCCCCTCCTGCAACTT
Cd36	GATGACGTGGCAAAGAACAG	CAGTGAAGGCTCAAAGATGG
Cpt1a	CTCAGTGGGAGCGACTCTTCA	GGCCTCTGTGGTACACGACAA
Cpt2	GCCCAAACCCCATTTTCTA	TAGGCAGAGGCAGAAGACAGCA
Acox1	GTATAAACTCTTCCCGCTCCTG	CACACAGTAGACGGCCTGAC
Acaa1a	TCAGGTGAGTGATGGAGCAG	CACACAGTAGACGGCCTGAC
Srebf1	AACCTCATCCGCCACCTG	TGGTAGACAACAGCCGCATC
Fas	TTGATGATTCAGGGAGTGGA	AGCAGATGAGTTGTTCTTGGAC
Scd1	CCACTCGCCTACACCAACG	GGGGTCCCTCCTCATCCT
Acc	TTGCCTATGAACTCAACAGCG	AGACCATTCCGCCCATCC
β-actin	CAGGCATTGCTGACAGGATG	TGCTGATCCACATCTGCTGG
